# How to Distinguish Patients with pSS among Individuals with Dryness without Invasive Diagnostic Studies

**DOI:** 10.1155/2018/1060421

**Published:** 2018-05-02

**Authors:** Agata Sebastian, Maciej Sebastian, Maria Misterska-Skóra, Patryk Woytala, Katarzyna Jakuszko, Piotr Wiland

**Affiliations:** ^1^Department of Rheumatology and Internal Medicine, Wroclaw Medical University, Borowska 213, 50-556 Wroclaw, Poland; ^2^Department of Minimally Invasive Surgery and Proctology, Wroclaw Medical University, Borowska 213, 50-556 Wroclaw, Poland; ^3^Department of Nephrology and Transplantation Medicine, Wroclaw Medical University, Borowska 213, 50-556 Wroclaw, Poland

## Abstract

In the course of pSS, inflammatory cell infiltration consists mainly of lymphocytes infiltrating exocrine glands, which leads to their impaired function. The characteristic feature is generalized dryness. The aim of this study was to attempt to answer the question whether it is possible to distinguish between patients with pSS and individuals with dryness caused by other pathologies without applying invasive studies. The study included 68 patients with pSS and 43 healthy controls with dryness. FS ≥ 1 was observed in 90% of patients with pSS (with or without dryness), and only in 23% of the control group (only with xerostomia). In the pSS group, anaemia (*p* = 0.0085), lymphocytopenia (*p* = 0.0006), elevated ERS (*p* = 0.001), higher RF titer, and ANA antibodies were noted. Configuration of anti-SSA + SSB + Ro52 antibodies was characteristic for the pSS group. Considering the clinical symptoms, statistically significant differences were noted between pSS patients and the control group in frequency (*p* = 0.02) and severity (*p* = 0.042) of fatigue, lymphadenopathy, major salivary gland involvement, and photosensitivity to UV light. In conclusion, invasive methods are pivotal in pSS diagnosis in this salivary gland biopsy. Chronic fatigue syndrome is more common in pSS patients and can be subjective distinguishing factor in the group of people with dryness.

## 1. Introduction

In the course of primary Sjögren's syndrome (pSS), inflammatory cell infiltration consists mainly of lymphocytes infiltrating exocrine glands, which leads to their impaired function. The characteristic feature is generalized dryness [1]. Inflammation may involve many organs evoking clinical symptoms depending on the exact location [2]. The disease develops slowly, and months can pass before the patient presents full spectrum of clinical symptoms. Insufficient treatment without inhibiting the autoimmune response leads to severe complications. The aim of this study was to attempt to answer the question whether it is possible to distinguish between patients with pSS and individuals with dryness caused by other pathologies without applying invasive studies.

## 2. Materials and Methods

The study included 68 patients (66 females and 2 males) diagnosed with pSS based on 2002 American-European Consensus Classification Criteria for pSS [3] after obtaining their informed consent (Bioethics Committee number 357/2010), who received medical care in the Department of Rheumatology and Internal Medicine between the years 2010 and 2013. Retrospectively, all patients met current (2016) classification criteria [4], which were unavailable at the time of the study.

The control group consisted of 43 individuals (5 males and 38 females), who had been observed and diagnosed for pSS due to dryness but finally were not diagnosed neither with pSS nor with any other rheumatic disorder.

The exclusion criteria encompassed the following: other autoimmune disease (e.g., rheumatoid arthritis, systemic lupus erythematosus, and systemic scleroderma), hepatitis C virus (HCV) and human immunodeficiency virus (HIV) infection, sarcoidosis, history of lymphoma, amyloidosis, hyperlipoproteinemia type V, graft-versus-host disease (GVHD), eosinophilia myalgia syndrome, history of head and neck irradiation, psychiatric and hypnotic drugs, uncontrolled hypertension, and uncontrolled diabetes mellitus.

We analyzed factors such as age, smoking, severity of dryness according to the EULAR Sjogren's Syndrome Patient Reported Index (ESSPRI) [5], fatigue assessed using the visual analogue scale (VAS) (0–10 cm) [6], intensity of inflammation in labial glands (LSB) [7] assessed by focus score (FS), organ pathology assessed using the EULAR Sjögren's syndrome disease activity index (ESSDAI) [8], laboratory tests such as anti-nuclear antibodies (ANA), extractable nuclear antigens (ENA), rheumatoid factor (RF), inflammatory parameters (erythrocyte sedimentation rate (ESR), C-reactive protein (CRP)), full blood count, and protein electrophoresis.

## 3. Statistical Analysis

For calculations, we used STATISTICA v. 9.0 software as well as Excel spreadsheet. In statistical analysis, we used Spearman's correlation rank coefficient (for paired variables with nonnormal distribution), Pearson's correlation coefficient (for paired variables with normal distribution), and linear regression. Mann–Whitney *U* test was used to verify differences between means for variables with normal nondistribution or nonhomogenous variances. Student *t*-test was used for differences between means for variables with normal distribution. Independence of quantitative and qualitative variables was using either nonparametric chi-squared or Fisher's test. All tests were conducted at the significance level of alpha = 0.05, *p* < 0.05.

## 4. Results

Mean age of patients with pSS was 51.2 (19–82) years. Mean time since the onset of symptoms to diagnosis was 7.5 years.

Mean age of healthy individuals was 51.1 (23–73) years. In the control group, total exclusion of connective tissue disorders since the onset of symptoms was 5.4 years.

### 4.1. Patients with pSS versus Control Group

In the studied group, mean severity of inflammation evaluated on pathology examination of LSB in FS was 2.2 in patients with pSS and 0.3 in the control group. FS ≥ 1 was observed in 90% of patients with pSS (with or without dryness), and only in 23% of the control group (only with xerostomia). Intensity of inflammatory infiltration expressed by the FS was much higher in patients with pSS (*p* < 0.00001, chi-squared test) (Table 1).

As far as laboratory test results are concerned, statistically significant differences (Mann–Whitney *U* test) between patients with pSS and the control group were noted in anaemia rate (*p* = 0.0085), lymphocytopenia (*p* = 0.0006), ERS level (*p* = 0.001), and in RF titer (*p* < 0.00001). No correlation between CRP level (*p* = 0.61) in pSS patients and the control group was found (13% versus 2%, resp.) (Table 1).

ANA antibodies were not found in 79% of healthy participants and in 19% of pSS patients. Statistical analysis (chi-squared test) showed that ANA antibodies are more common in pSS patients compared to the control group, and the difference was statistically significant (*p* < 0.00001).

Antibodies most commonly detected in pSS were anti-SSA (82% of patients), anti-Ro52 (70% of patients), and anti-SSB (69% of patients). Configuration of all three specific antibodies anti-SSA + SSB + Ro52 was the most common (among 54% of patients), while anti-SSA + SSB combination was observed in 15% of patients and anti-SSA + Ro52 in 13% of patients. None of the pSS patients was positive for anti-SSA or anti-SSB antibodies only, without any other specific antibodies (Table 2).

### 4.2. Clinical Symptoms in pSS Patients versus Control Group

Mean age of pSS patients at the onset of symptoms was 51 ± 14.39 years (minimum 12 and maximum 71). In the control group, mean age of participants at the onset of dryness was 42 ± 11.42 (minimum 20 and maximum 72) years.

Mean time between pSS diagnosis and first clinical symptoms was 7.5 years (SD 7.5). Mean time needed for exclusion of pSS in the control group was 5.4 years (SD 4.4).

Considering severity of xerophthalmia, xerostomia, and vaginal dryness, as well as subjective sensation of fatigue, statistically significant differences (Mann–Whitney *U* test) were noted between pSS patients and the control group in frequency (*p* = 0.02) and severity (*p* = 0.042) of fatigue. Female patients with pSS reported vaginal dryness more often (*p* = 0.002); however, the intensity of this symptom was comparable in both groups. pSS patients more frequently reported more intensive fatigue affecting daily activity (5.46 cm on VAS scale on average). Mean severity scores for dryness symptoms were as follows: xerophthalmia 4.47 cm for the pSS group and 4.28 cm in the control group (*p* = 0.96), xerostomia 4.76 cm for the pSS group and 3.77 cm in the control group (*p* = 0.12), and vaginal dryness in women: 3.03 cm for the pSS group and 2.82 cm in the control group (*p* = 0.64). Differences in severity of dryness symptoms between pSS patients and the control group were not observed.

Table 3 presents frequency of clinical symptoms in patients with pSS and in the control group. Physical examination and history taking revealed that skin lesions, peripheral joint swelling, as well as major salivary gland involvement (examined physically or by diagnostic ultrasound), swollen lymph nodes, and UV-light photosensitivity were statistically more common in patients with pSS.

Skin lesions were in 19 (30%) patients with pSS observed as erythema, urticaria, purpura on lower extremities, livedo reticularis, erythematous, and exfoliative lesions. Skin lesions in the control group never took the form of purpura, but rather erythema only, and were observed significantly less often than in pSS group (19 versus 2 patients). Mean age in pSS population with skin changes was 47 (SD 14) years, while the mean time needed for final diagnosis of pSS was 11 years (SD 10); patients without skin lesions tended to be older (mean age of 53, SD 13; *p* = 0.1), and the time needed for the final diagnosis of pSS was shorter (6 years on average, SD 4). No correlation between skin lesions and CRP level (*p* = 0.9), ESR (*p* = 0.1), FS (*p* = 0.05), leukocyte count (*p* = 0.1), RF titer (*p* = 0.3), gamma globulin level (*p* = 0.06), severity of xerophthalmia (*p* = 0.3), and xerostomia (*p* = 0.9), as well as the presence of anti-SSA, anti-SSB, and anti-Ro52 specific antibodies, was observed. Among pSS patients with hypergammaglobulinemia, skin lesions were more common (66% versus 33%) and the difference was statistically significant.

The chest HRCT scan revealed changes in the lung tissue in the course of 29% of all examined patients with pSS. The most common lesions were fibrosis, enlarged mediastinal lymph nodes, and nodes which was previously reported in other publication [9]. Chest high-resolution computed tomography (HRCT) was not performed in the control group due to the lack of indications for this type of diagnostic study (no pulmonary involvement).

### 4.3. Lymphadenopathy

**Figure 1:**
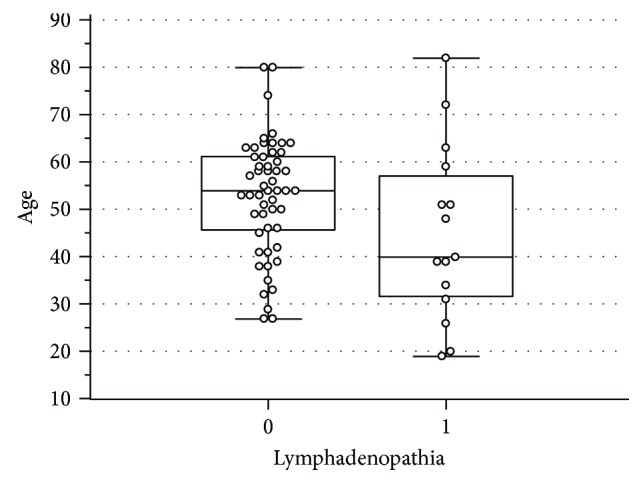
Correlation between the age of pSS patients and lymphadenopathy.

Peripheral lymphadenopathy was reported in 22% of pSS patients. Lymphadenopathy occurred more frequently among younger patients (*p* = 0.04) (Figure 1) (mean age of 45 years SD 18 versus 53 years SD 12). Time to final diagnosis of pSS in patients with lymphadenopathy was 9 years on average (SD 6 years), while it took 7 years (SD 8) without that symptom.

No correlation between lymphadenopathy and CRP level (*p* = 0.5), ESR (*p* = 0.2), FS (*p* = 0.2), and leukocyte count (*p* = 0.5) was found. In patients with lymphadenopathy, RF titer was higher and the difference was statistically significant (*p* = 0.003). Mean RF titer in patients with lymphadenopathy was 110 IU/ml, while in patients who did not present that symptom was 70 IU/ml.

**Figure 2:**
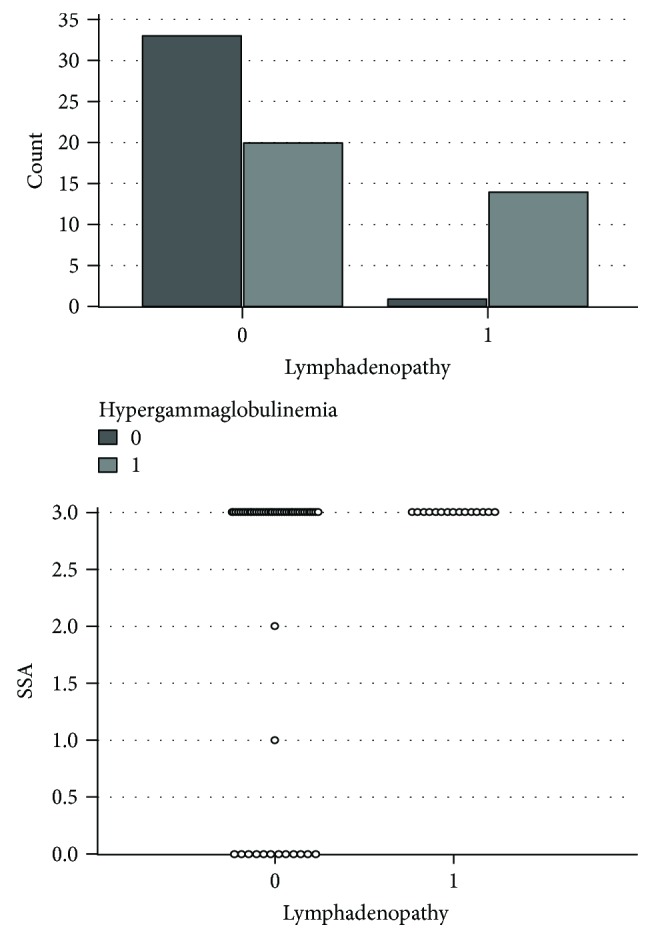
Lymphadenopathy rate in patients with hypergammaglobulinemia and its correlation with anti-SSA antibodies.

Among patients with pSS and lymphadenopathy, elevated level of gamma globulins (2.1 g/dl, SD 0.8) was noted, compared to pSS patients without lymphadenopathy, and that symptom was more prevalent in patients with hypergammaglobulinemia (*p* = 0.0004), the difference being statistically significant (Figure 2).

In patients with lymphadenopathy, high titer of anti-SSA specific antibodies (3.0 versus 2.2; *p* = 0.02) was observed compared to patients who did not present this symptom (Figure 2).

All pSS patients with lymph node enlargement tested positive for specific anti-SSA antibodies, while the results were positive in 77% of patients without this symptom; however, the difference was not statistically significant (*p* = 0.05).

No correlation between the presence or titer of anti-SSB specific antibodies and lymphadenopathy was observed (*p* = 0.06; *p* = 0.1).

### 4.4. Musculoskeletal Involvement

Arthralgia was reported by 47% of patients at the onset of the disease and by 70% during the entire course of observation. In the control group, 65% reported arthralgia. Arthritis was observed in 30% of pSS patients (in 15% at the onset of the disease) and in 5% of healthy participants. Pain sensation mostly affected minor joints of the hand and knees. Inflammation was located within minor joints of the hand and ankles. Also, muscle pain was more prevalent in pSS patients compared to the control group (13% versus 5%, resp.).

Joint swelling was observed in 30% of pSS patients (20 patients). The mean age of patients with joint swelling was 47 (SD 13) years, and the mean time required for final diagnosis of pSS was 9 years (SD 9). In patients with no musculoskeletal involvement, the mean age was 52 (SD 14) years and the mean time required for final diagnosis of pSS was 7 years (SD 7). No correlation was found between inflammation of the joints and age (*p* = 0.1), CRP level (*p* = 0.6), ESR (*p* = 0.6), FS (*p* = 0.4), leukocyte count (*p* = 0.7), RF titer (*p* = 0.8), gammaglobulin level (*p* = 0.6), severity of xerophthalmia (*p* = 0.6), and xerostomia (*p* = 0.2) as well as the presence of anti-SSA (*p* = 0.8), anti-SSB (*p* = 0.1), and anti-Ro52 (*p* = 0.2) antibodies.

### 4.5. Time Required for Final Diagnosis of pSS

The shortest time required to make the final diagnosis of pSS was observed in the case of pulmonary involvement (7 years SD 6), and it was the longest for skin lesions (11 years SD 10). Distribution of time to final diagnosis depending on symptoms in pSS patients is shown in Table 4.

## 5. Discussion

In clinical practice, most patients with pSS report dryness. However, symptoms are not specific for a single disease. On the other hand, dryness observed in pSS may develop after months, while other symptoms of pSS can constitute primary manifestation, such as musculoskeletal and neurological symptoms or lymphomas. Due to this variety of clinical presentation, the time needed for the right diagnosis is longer and takes about 3 to 8 years since the onset of symptoms [10]. In the studied group, the time was 7.5 years on average (92 months) and was the shortest in the case of pulmonary involvement (7 years, SD 6 years) and the longest for skin presentation (11 years, SD 10 years). However, in individuals with rheumatologic disorders excluded, the time needed to exclude autoimmune basis of dryness was 5.4 years on average.

Xerophthalmia and xerostomia in both pSS patients and the control group occurred at a similar rate (79% versus 79% and 81% versus 77% accordingly). As the primary symptom, pSS patients reported xeropthalmia (54%), while healthy individuals reported xerostomia (49%). The results were similar to those reported by other authors, who summarized the frequency of each symptom in pSS [3, 11]. Due to the fact that dryness can be caused by many factors and disorders, diagnosis of pSS is not possible solely based on the presence and severity of dryness. On the one hand, patients are observed for other diseases and they tend to ignore dryness symptoms. On the other hand, elderly patients reporting peripheral arthralgia and dryness are difficult to diagnose. In that group, more patients report dryness, which is often caused by physiology and hormonal disturbances [12].

Considering severity of xerophthalmia, xerostomia, and vaginal dryness as well as subjective feeling of excessive fatigue, there was a significant difference in intensity and rate of fatigue between pSS patients and healthy individuals. Patients with pSS complained about more pronounced fatigue impairing their daily activity compared to the control group. However, no difference in severity of dryness was observed between patients and healthy individuals, except for vaginal dryness in females, which was more frequent in pSS patients. Nevertheless, its severity (VAS scale) was comparable to healthy individuals. Therefore, based solely on dryness, it is impossible to distinguish between symptoms of pSS and other causes in an outpatient setting.

In etiopathogenesis of fatigue in pSS, psychological factors, autoimmune response, hormonal dysfunction, cytokines, and viral infections can all play an important role [13]. Ramos-Casals et al. showed that excessive fatigue relating to pSS occurs in 70–80% of patients and often interferes with work [14]. In the studied group, the rate of fatigue was even higher reaching over 90%, which indicates necessity to use fatigue-assessing clinical scales (e.g., FACID and VAS) in everyday rheumatologic assessment of pSS patients. No relationship between severity of fatigue reported by patients and laboratory test results or lymphocytic infiltration in labial glands was found. Other less specific general symptoms in pSS and related to fatigue included sleep disturbances, anxiety, and depression; the frequency of such symptoms in pSS accounts to 15%, 20%, and 40%, respectively [14].

Moreover, in about 50% of pSS patients, musculoskeletal pain can be observed [14, 15]. In our study, musculoskeletal pain was split into muscle and peripheral joint pain. It turned out that muscle pain is far less common compared to joint pain in both pSS patients and the control group. None of the symptoms was pathognomonic for the studied group. Thus, it is incorrect to state that every person with dryness and musculoskeletal symptoms must suffer from pSS. It is worth noting that a substantial percentage of individuals reported arthralgia despite exclusion of connective tissue disease and rheumatoid arthritis after full diagnostic work-up. It makes diagnosis of pSS more difficult, being one of the common but noncharacteristic symptoms.

Similar conclusions were drawn by Hackett et al. in their study. They showed the influence of pSS on daily activity in patients compared to healthy individuals with the same age and sex. It turned out that pSS patients complained about pain more frequently compared to healthy individuals (*p* < 0.0001) in a statistically significant manner; they also more often reported depression (*p* < 0.0001). Also, lower Health Assessment Questionnaire (HAQ scores) were noted (*p* = 0.002) [16].

In our study, musculoskeletal symptoms were observed at a considerable frequency in pSS patients, among whom 47% complained about peripheral arthralgia at the beginning of the disease and 70% during the entire observation period. In the control group, pain was commonly observed as well. The participants differed as to the rate of peripheral joint inflammation, which was observed in 30% of pSS patients (including 15% at the onset of the disease). Among healthy individuals, widened joint contours were observed only in 5% of participants and were associated with osteoarthritis. Pain sensation in pSS patients affected mostly minor joints of the hand and knees. Inflammation involved mostly minor joints of the hand and ankles. No correlation between peripheral joint involvement and hypergammaglobulinemia, intensity of inflammation assessed by focus score, and, interestingly, RF titer was found. It should be noted that RF itself is a relatively common occurrence found in 40–70% of pSS patients [17]. In our study, RF was present in 70% of pSS patients. RF was statistically less frequent in healthy individuals. Therefore, in differential diagnosis of musculoskeletal pain and suspicion of pSS, RF testing should be carried out routinely.

Moreover, based on a history taking and physical examination, features distinguishing individuals with dryness and pSS patients include skin lesions typical for pSS, photosensitivity to UV light, large salivary gland involvement, and peripheral lymphadenopathy (excluding infections and hematological disorders). Special attention should be given to those symptoms in diagnosis of pSS, despite the fact that not all of them (UV-light photosensitivity) are included in the disease activity scales (ESSDAI). Peripheral lymphadenopathy alone is statistically more frequent in younger individuals, and it correlates with anti-SSA antibodies and hypergammaglobulinemia.

Among invasive diagnostic studies, specific ENA antibodies and infiltration assessment on FS according to Fisher's protocol [6] remain characteristic and irreplaceable, and so they have been included in all classification criteria for pSS so far [3, 4, 18]. In the studied group, anti-SSA antibodies proved to be characteristic for pSS, and it was more commonly found than SSB antigen. It has been confirmed by results published by Baer et al. [19] and explains why anti-SSB antibodies have not been included in new current classification criteria for pSS [18]. However, the combination of anti-SSA, anti-SSB, and anti-Ro52 antibodies in the same patient increases probability of pSS diagnosis as shown in our study. Interestingly, the diagnosis rate of pSS was lower in patients, and in some cases, anti-Ro52 were not found. None of the participants was found with both anti-SSA and anti-SSB antibodies without coexisting other specific antibodies. It might be related to a low number of the studied group; nevertheless, it requires further analysis on a larger cohort. It should be remembered that specific antibodies may be predicting factors of developing pSS later [20].

Being unable to distinguish pSS patients from individuals reporting dryness solely based on xerostomia with coexisting risk factors of pSS (skin lesions, swollen salivary glands, peripheral lymphadenopathy, photosensitivity to UV light, and fatigue), it is necessary to perform LSB with pathology assessment according to pSS protocol [6]. As shown in our study, which confirmed the results of other studies from recent years [21], it is the key examination in this group of patients. Despite its invasiveness, the procedure itself is simple and lasts about 20 minutes including patient preparation; it does not require suturing and is associated with low risk of complications. Positive result narrows down the differential diagnosis and is rarely found in healthy individuals [22]. Therefore, pathology assessment of minor salivary glands has been included in classification criteria for pSS for years and plays a crucial role [3, 4].

As shown in our study, diagnosis of pSS is prolonged by a few years, 7.5 on average. The least time required for final diagnosis of pSS was observed in the case of pulmonary involvement, and it was the longest for skin lesions. Similarly, individuals with dryness symptoms were observed for 5 years on average before pSS was excluded as the cause of the symptoms. It may be due to the fact that clinical symptoms of pSS develop with time. The course of the disease is long-standing and insidious, noncharacteristic for this disease only, and dryness itself is quite common in general population. Symptoms of pSS usually develop gradually, which results in kind of becoming accustomed to, for example, dryness; physicians diagnose other diseases even in the advanced form of pSS. Thus, pSS patients are referred to many specialists before visiting rheumatologist after months, who can either exclude or confirm pSS only after invasive studies (ANA/ENA antibodies, biopsy, and LSB), which remain the gold standard.

## Figures and Tables

**Table 1:** Values of laboratory test results, immunological markers, and severity of dryness and fatigue in patients with pSS and in the control group.

Study group	Number of patients/%	Number of patients/%	Minimum	Minimum	Maximum	Maximum	*p* value
	pSS	Control	pSS	Control	pSS	Control	
ANA > 1 : 320 *n*/%	55/81%	9/21%	1 : 320	1 : 320	1 : 10000	1 : 320	<0.000

CRP mean value	2.38 ± 3.71	2.13 ± 2.84	0.19	0.00	24.38	18.30	
CRP > 5 mg/dl *n*/%	9/13%	1/2%					0.047

Focus-score LSB mean value	2.22 ± 1.35	0.37 ± 0.93	0.00	0.00	4.00	4.00	
FS ≥ 1 *n*/%	61/90%	10/23%					<0.001

Hemoglobin level mean value	12.99 ± 1.78	13.52 ± 1.23	10.40	11.00	24.80	16.50	
Anaemia *n*/%	15/22%	3/7%					0.029

WBC mean value	5.43 ± 2.09	5.99 ± 1.61	1.95	3.73	14.40	10.10	
WBC < 4 tys. *n*/%	19/28%	1/2%					0.0003

Lymphocyte mean value	1.58 ± 1.16	1.79 ± 0.46	0.48	0.70	9.99	2.70	
Lymphopenia *n*/%	39/57%	6/14%					<0.001

Oral dryness mean value	4.76 ± 2.99	3.77 ± 2.93	0.00	0.00	10.00	10.00	0.12
>0 in VAS *n*/%	60/88%	32/74%					0.05

Dry eye mean value	4.47 ± 2.69	4.28 ± 2.70	0.00	0.00	10.00	8.00	0.96
>0 in VAS *n*/%	62/91%	36/84%					0.18

Vaginal dryness mean value	3.03 ± 2.73	2.82 ± 2.94	0.00	0.00	9.00	9.00	0.64
>0 in VAS *n*/%	41/62%	11/29%					<0.000

Fatigue mean value	5.46 ± 2.37	3.84 ± 2.65	0.00	0.00	10.00	8.00	0.04
>0 in VAS *n*/%	67/98%	35/81%					0.002

ESR mean value	31.51 ± 24.77	16.02 ± 11.62	6.00	1.00	103.00	53.00	0.001
ESR > 20 mm/hr *n*/%	39/57%	10/23%					0.0004

RF mean value	79.32 ± 128.67	12.31 ± 25.94	0.00	0.00	801.00	171.80	
RF > 14 IU/ml *n*/%	50/73%	7/16%					<0.000

*n*: number of patients; normal value: ANA: antinuclear antibodies < 1 : 320 (EUROIMMUN Hep-20-10/liver Monkey set); CRP: C-reactive protein < 5 mg/dl; LSB: labial salivary gland biopsy; haemoglobin: 12–16 g/dl in women, 14–18 g/dl in men; WBC: white blood cells 4–10 k/*μ*l; lymphocytes: 1.5–3.5 k/*μ*l (in complete peripheral blood count); VAS: visual analogue scale (0–10 cm); ESR: erythrocyte sedimentation rate 3–15 mm/h; RF: 0–14 IU/ml.

**Table 2:** Detailed distribution of anti-SSA, anti-SSB, and anti-Ro52 specific antibodies and their titers (luminous intensity 0–3) in pSS patients.

Anti-SSA—number of patients/%	Anti-SSB—number of patients/%	Anti-Ro52—number of patients/%
Titer 3+—54 patients/79%	Titer 3+—37 patients/54%	Titer 3+—44 patients/65%
Titer 2+—one patient	Titer 2+—6 patients/9%	Titer 2+—0 patients
Titer 1+—one patient	Titer 1+—3 patients	Titer 1+—3 patients
	Titer 0.5+—one patient	Titer 0.5+—one patient

Only anti-SSA—0 patients	Only anti-SSB—0 patients	Only anti-Ro52—3 patients/4%
anti-SSA + SSB—10 patients/15%	anti-SSA + SSB—10 patients/15%	anti-SSA + Ro52—9 patients/13%
anti-SSA + Ro52—9 patients/13%	anti-SSB + Ro52—0 patients	anti-SSB + Ro52—0 patients
anti-SSA + SSB + Ro52—37 patients/54%	anti-SSA + SSB + Ro52—37 patients/54%	anti-SSA + SSB + Ro52—37 patients/54%

For ANA testing, the EUROIMMUN Hep-20-10/liver monkey set was used. For determination of antigen specificity of the anti-nuclear antibodies, Anti-Ena Profile Plus 1 Euroline immunoblotting set was used.

**Table 3:** Clinical features in pSS patients and in the control group.

Clinical presentation	Primary Sjögren's syndrome (number of patients/%)	Control group (number of patients/%)
Arthralgia	48/70%	28/65%
Arthritis^∗^	20/30%	2/5%
Large salivary gland involvement^∗^	33/48%	4/9%
Raynaud's syndrome	8/12%	2/5%
Bone marrow infiltration	1/1%	0
Alopecia	2/3%	0
Muscle soreness	9/13%	2/5%
Hearing loss	6/9%	3/7%
Abnormal chest HRCT	18/26%	Not performed
Gastrointestinal	7/10%	2/5%
Polyneuropathy	7/10%	1/2%
Peripheral lymphadenopathy^∗^	15/22%	0
UV-light photosensitivity^∗^	7/10%	0

^∗^Statistically significant. Bone marrow infiltration was defined as an abnormal percentage of plasmocytes on bone marrow biopsy (>3.5% plasmocytes). Gastrointestinal involvement included pancreatitis, enlarged lymph nodes on imaging, hepatomegaly and/or splenomegaly, diarrhea (watery stools, >200 g/d, >3 stools daily), weight loss, and nonspecific abdominal pain. Insomnia: difficulty of falling asleep or early waking up lasting for more than 2 weeks; peripheral lymphadenopathy: swollen lymph nodes > 1 cm on physical examination except for inguinal lymph nodes, where the cut-off size is >2 cm.

**Table 4:** Distribution of time to final diagnosis of pSS depending on symptoms.

Clinical presentation	Time required for final diagnosis of pSS	Standard deviation (SD)
Abnormal chest HRCT	7	6
Major salivary gland involvement	10	9
Lymphadenopathy	9	6
Skin lesions	11	10
Peripheral arthritis	9	9
